# Neurons for Numerosity: As Quantities Increase, So Does the Neuronal Response

**DOI:** 10.1371/journal.pbio.0050226

**Published:** 2007-07-24

**Authors:** Liza Gross

Humans stand apart from other species in our ability to grasp higher math, but evolution laid the groundwork for numerical cognition long before ancient mathematicians drew symbols on clay tablets. The capacity for numerical cognition has been demonstrated in great apes, monkeys, numerous birds, dolphins, dogs, lions, rats, and raccoons, a list apparently restricted more by experimental inquiry than by inherent limitations in animals. In the wild, animals use this nonverbal ability to approximate number, called numerosity, to guide decisions on where to forage, when to flee from predators, and whether to fight intruders.

Evidence from humans and monkeys suggests that the intraparietal sulcus (IPS), a deep groove along the lateral surface of the parietal cortex, plays an important role in number processing. Injury to the parietal cortex impairs patients’ comprehension of number, and studies on rhesus monkeys identified parietal neurons selectively tuned to specific cardinal numbers. However, previous studies found that only a relatively small percentage of parietal neurons were tuned to numbers—finding a much higher percentage of number-sensitive neurons in the prefrontal cortex. The responses of these number-specific neurons reflected specific cardinal values rather than accumulated quantity; however, and the studies did not entirely rule out the interference of several task-related factors.

In a new study, Jamie Roitman, Elizabeth Brannon, and Michael Platt report that neurons in the lateral intraparietal area (LIP) respond specifically to numerical quantity. Although such “accumulator” neurons—which encode approximate quantity rather than number identity—have been proposed by computational models of number processing, evidence of their existence had been inconclusive.

Previous studies have linked LIP activity with experimental tasks that rely on synthesizing spatial, temporal, and reward information, suggesting that LIP neurons integrate this information to guide behavior. These findings suggested that LIP neurons could theoretically integrate, or accumulate, information on numerical quantity.

**Figure:**
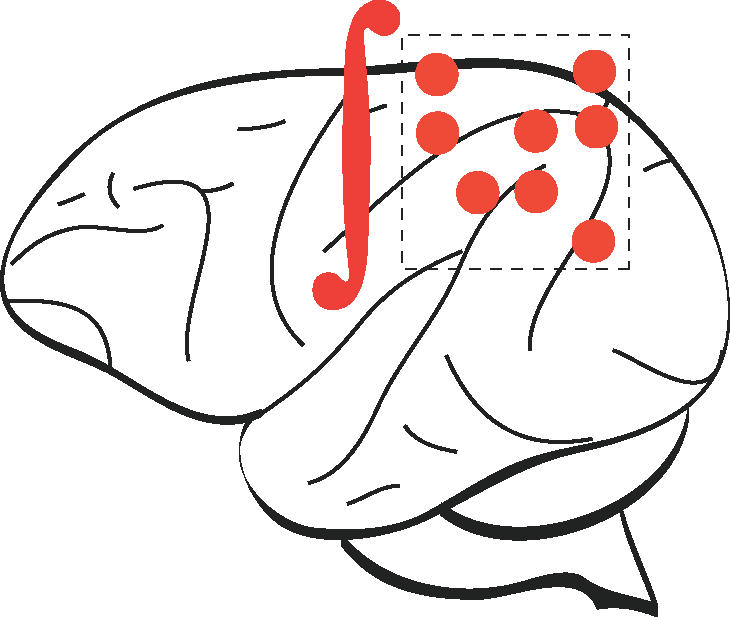
Neurons in the lateral intraparietal area of the monkey brain have been shown to integrate information about space and time. A new study asks whether they also “add up” numerical quantity.

Because LIP neurons can respond to multiple stimuli and tasks, identifying the neural mechanisms of complex cognitive traits like numerosity is especially challenging. Controlling for confounding factors becomes even more onerous when the function of the brain area under investigation—like the LIP—has not been firmly established. Still, it’s clear that many factors could modulate LIP neuron activity, including expectation of reward, paying attention to stimuli in certain parts of space, and motor planning (for eye movements, or saccades).

To minimize the amount of training required (which can also confound results) and reduce the influence of task-related factors on neural activity, Roitman et al. studied the activity of single LIP neurons in two monkeys performing a task that did not require explicit judgments about numerosity. This task also exploited the fact that individual LIP neurons respond selectively to visual targets placed only within a specific spatial window, called the spatial receptive field.

To ensure that numerosity stimuli would be presented within each neuron’s receptive field, the researchers recorded from individual neurons as monkeys performed what’s called a delayed-saccade task, traditionally used to stimulate activity in these neurons. Monkeys were trained to fix their gaze on a central focal point and then got a juice reward for shifting their gaze to a peripheral target after the focal point disappeared. By placing the targets in different locations throughout the monkey’s visual field, the researchers were able to establish the edges of the area that triggered a neural response in each LIP neuron.

With the receptive fields mapped, the researchers moved on to the implicit numerical task. They displayed numerosity stimuli (sets of two, four, eight, 16, or 32 dots) within the receptive fields and placed the saccade targets outside, so neural activity associated with motor planning was not a factor. Unlike previous studies, this study used a wide range of values to distinguish between responses that were accumulative in nature and responses that were tuned to specific cardinal values. The researchers controlled for reward expectation by presenting a standard number of dots, say eight, with a standard-size reward in half of the trials and then presenting a deviant number of dots (randomly jumping between two, four, 16, and 32) with a larger reward in the other half. Varying the standard and deviant numerosities across trial blocks decoupled reward expectation from specific numerosities.

For this task, monkeys fixated on a central point while a saccade target appeared in a random location on the opposite side of the computer screen from the receptive field. After a short delay, the numerosity array appeared within the receptive field. When the fixation point disappeared, the monkey shifted his gaze to the saccade target for his juice reward. Because the numerosity stimulus appeared within the classical receptive field (and the saccade target appeared in the opposite direction to control for motor-related neural activity), elevated neural activity should reflect numerosity, if LIP neurons integrate quantity.

And that’s what happened. The numerosity arrays triggered a large neural response, with over half of the LIP neurons responding monotonically—their activity either progressively increased or decreased with the number of elements in the array. Most of the neurons showed preferences for either the smallest or largest arrays tested, and their firing rate depended on number, but not absolute value.

These results indicate that integrator neurons in the LIP accumulate quantity and suggest that the brain uses distinct mechanisms to judge accumulated magnitude and specific cardinal values, as computational models propose. Since accumulating quantity is a critical step in counting, neurons tuned to specific cardinal values may rely on LIP neurons to derive those numbers. The coincident representation of space and number in LIP neurons may also help explain why patients with parietal lobe damage from stroke or Gerstmann syndrome can no longer distinguish left from right or make simple calculations.

